# Ectopic expression of microRNA-155 enhances innate antiviral immunity against HBV infection in human hepatoma cells

**DOI:** 10.1186/1743-422X-8-354

**Published:** 2011-07-18

**Authors:** Chenhe Su, Zhaohua Hou, Cai Zhang, Zhigang Tian, Jian Zhang

**Affiliations:** 1 School of Pharmaceutical Sciences, Shandong University, Jinan 250012, China

**Keywords:** miR-155, HBV, anti-virus, hepatoma cells, innate immunity

## Abstract

**Background:**

Host innate antiviral immunity is the first line of defense against viral infection, and is precisely regulated by thousands of genes at various stages, including microRNAs. MicroRNA-155 (miR-155) was found to be up-regualted during viral infection, and influence the host immune response. Besides, the expression of miR-155, or its functional orthologs, may also contribute to viral oncogenesis. HBV is known to cause hepatocellular carcinoma, and there is evidence that attenuated intracellular immune response is the main reason for HBV latency. Thus, we assume miR-155 may affect the immune response during HBV infection in human hepatoma cells.

**Results:**

We found that ectopic expression of miR-155 upregulated the expression of several IFN-inducible antiviral genes in human hepatoma cells. And over-expression of miR-155 suppressed suppressor of cytokine signaling 1 (SOCS1) expression and subsequently enhanced signal transducers and activators of transcription1 (STAT1) and signal transducers and activators of transcription3 (STAT3) phosphorylation. We further demonstrate that ectopic expression of miR-155 inhibits HBV X gene expression to some extent *in vitro*.

**Conclusion:**

MiR-155 enhances innate antiviral immunity through promoting JAK/STAT signaling pathway by targeting SOCS1, and mildly inhibits HBV infection in human hepatoma cells.

## Background

MicroRNAs (miRNA) are a class of highly conserved short noncoding RNAs originate from genome of eukaryotic organisms and even kinds of virus. MiRNAs have emerged as a major class of post-transcriptional gene-expression regulators and are involved in a wide variety of biological processes. They regulate target gene expression mainly through imperfectly base pairing with the 3' -untranslated regions (3'-UTRs) of target mRNAs in animals, preventing protein accumulation by inducing mRNA degradation or suppressing translation [1,2].

Recent researches demonstrate that many miRNAs, such as miR-146a, miR-155 and miR-223, play important roles in innate immune response at various phases in vertebrates [3]. In order to eliminate virus immediately after infection, host-encoded miRNAs can directly interfere in virus replication. Meanwhile, virus-encoded miRNAs can not only evolve to regulate viral gene expression to accommodate life cycle and maintain latency [4], but also affect cellular gene expression by directly participating in host gene expression or by mimicking cellular miRNAs to hijack in unclear cellular regulatory networks. So, research on the roles of miRNA is an important pathway to reveal the "fighting" between pathogenic microorganism and their hosts.

Among all miRNAs discovered in Homo sapiens in miRBase Release 17, miR-155 is considered as one of the typical multifunctional miRNAs. MiR-155 is first found within the B cell integration cluster (BIC) on chromosome 21 in human genome. The genomic structure of human BIC consists of three exons, and miR-155 is located within the third exon [5]. To date, increasing evidence reveals that miR-155 is involved in numerous physiological and pathological processes including innate and adaptive immunity, inflammation and tumorigenesis [6]. Moreover, miR-155 was found to be induced by poly (I:C) and other TLR ligands through either MyD88 or TRIF dependent signaling pathways, and by IFN-β and several cytokines such as IL-1β through TNF-α signaling.

Viruses may downregulate the expression of certain cellular miRNAs that are harmful for viral movement and may also upregulate some miRNAs that are rational for the virus. Although the benefit of miR-155 expression to viruses remains largely unknown, the expression of miR-155, or its functional orthologs, may contribute to viral oncogenesis, like KSHV [7] and MDV-1 [8] encoding a viral miR-155 ortholog while EBV instead induces expression of endogenous miR-155. However, till now, there are only a few reports about the roles of miR-155 in viral infection.

HBV, which causes acute and always chronic HBV infection and leads to hepatoma fibrosis, cirrhosis and eventually hepatocellular carcinoma [9], has infected more than 350 million people and become a main threaten to public health across the world, especially in developing countries [10]. Modern immunology believed that impaired immune system, both intrinsic immune response and classical humoral/cellular immunity is the major reason for HBV latency, chronicity and reactivity, and many achieves have been made to explain the molecular mechanism upon this topic[11].

Based on all the outcomes have been made in this field, we become interested in the roles of miRNA in hepatocyte-HBV interactions. Though miR-155 level was very low in normal hepatocytes and several hepatoma cells lines, but it can be upregulated in certain pathological process, such as tumorigenesis. Considering that HBV can promote carcinomatous change in hepatocyte, it can be expected that miR-155 may be a unique molecular in HBV pathopoiesis. But until now there is no report about the role of miR-155 on the innate antiviral immunity in human hepatocytes or hepatoma cells.

In the present study, miR-155 was over-expressed in human hepatoma cells (HepG2, H7402), and we found that SOCS1 expression was suppressed and subsequently the phosphorylation STAT1 and STAT3 were enhanced, which resulted in the induction of IFN-inducible genes expression. Additionally, over-expression of miR-155 slightly increased the proliferation ability of HepG2 cells, while exhibited mild anti-HBV effect in human hepatoma cells. These findings reveal a new intricate physiological interplay between miRNAs and HBV replication.

## Results

### Over-expression of miR-155 in human hepatoma cells

**Figure 1:**
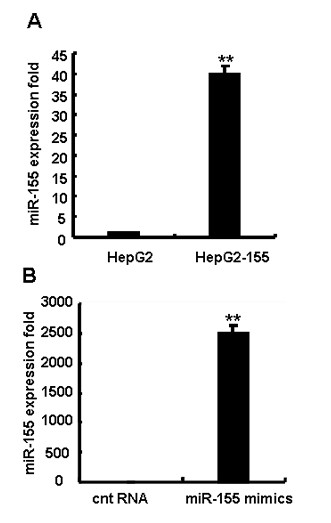
**Over-expression of miR-155 in human hepatoma cells**. (A) qRT-PCR analysis of miR-155 levels in miR-155 gene-modified HepG2 (HepG2-miR155) cells and parental HepG2 cells. (B) 50 nM miR-155 mimics or control RNA (cnt RNA) were transfected into HepG2 cells. qRT-PCR analysis was performed 48 h after transfection. Data shown are means ± SD from at least three separate experiments. (*P < 0.05, **P < 0.01 by comparison with control cells).

To investigate the role of miR-155 in antiviral immune response in human hepatoma cells, we used two methods to over-express miR-155 in human hepatoma cells [12,13]. First, the plasmid pcDNA3-hsa-miR-155 was transfected into HepG2 cells using Lipofectamine 2000, and these cells were selected by G418 for two weeks. Levels of miR-155 in transfectant HepG2 cells (HepG2-miR155) were evaluated by qRT-PCR. As shown in Figure 1A, miR-155 level in HepG2-miR155 cells was significantly higher than that in parental HepG2 cells (~ 40 folds) (*P *< 0.01). Next, miR-155 mimics or control RNA (cnt RNA) were transiently transfected into HepG2 cells. As shown in Figure 1B, the level of miR-155 in HepG2 cells transfected with miR-155 mimics was much higher (~ 2500 folds) than that in HepG2 cells transfected with control RNA (*P *< 0.01).

### miR-155 slightly increased the growth of human hepatoma cells

**Figure 2:**
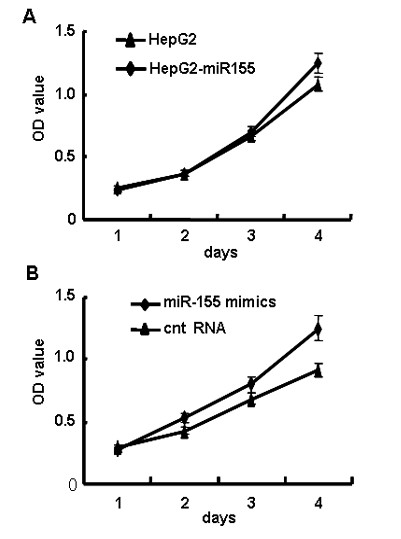
**miR-155 slightly increased the growth of human hepatoma cells**. (A) HepG2 and HepG2-miR155 cell growth were determined by MTT assay. (B) HepG2 cells were transfected with 50 nM miR-155 mimics or control RNA followed by MTT assay 6 h after transfection. These experiments were repeated at least 3 times.

MiR-155 has been reported to be over-expressed in many solid tumor cells and functions as 'oncomir' [6,14]. So, we identified whether miR-155 could regulate human hepatoma cell proliferation in vitro. Until cultured for 4 days, it showed a slight increase in the proliferation of miR-155 over-expressed HepG2 cells, especially HepG2 cells transfected with miR-155 mimics, when compared to that of control cells (Figure 2A and 2B), indicating that miR-155 displays different roles in diverse cells.

### Upregulation of IFN-inducible antiviral genes by miR-155 over-expression in human hepatoma cells

**Figure 3:**
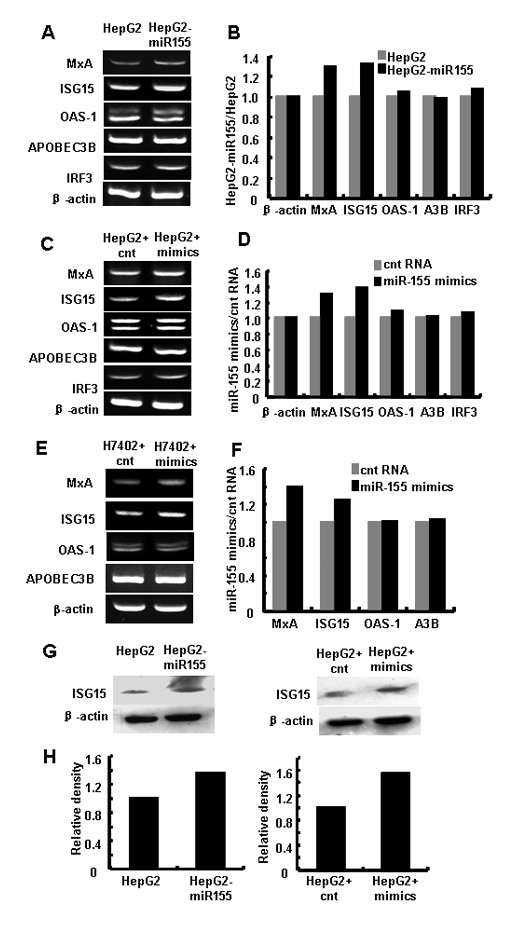
**Over-expression of miR-155 enhanced IFN-Inducible genes expression in human hepatoma cells**. HepG2 cells, HepG2-miR155 cells or HepG2 cells and H7402 cells transfected with 50 nM miR-155 mimics/control RNA were harvested. As described in Materials and Methods, (A, C) RT-PCR analysis was performed to evaluate IFN-inducible genes. (B, D) Relative density analysis of IFN-induced gene mRNA levels. (E, F) RT-PCR analysis and relative density analysis of IFN-inducible genes in H7402 cells 48h after transfection. (G, H) Western blot analysis of ISG15 levels in HepG2 cells treated as above. One representative of three independent experiments was shown. (*p < 0.05 by comparison with control cells).

The type I interferon dependent cytokines are recognized as crucial components during the innate immune response and the first line of antiviral defense. In the studies of gene knockout mice, ISG15, Mx proteins (MxA), 2'-5' oligoadenylate synthetase (OAS) and APOBEC3 family, such as APOBEC3B (A3B) have been validated as antiviral effectors [15-17]. Therefore, the expression of MxA, ISG15, OAS-1, A3B and interferon regulatory factor3 (IRF3) in HepG2 cells were evaluated by RT-PCR (Figure 3A and 3C). We found that the mRNA levels of MxA and ISG15 in miR-155 over-expressed HepG2 cells were increased about 30% and 40% respectively, while the levels of A3B, OAS1 and IRF3 were not significantly changed (Figure 3B and 3D). Similar results were observed in another human hepatoma cell line H7402 (Figure 3E and 3F). Western blot analysis showed that ISG15 protein levels were increased as well (Figure 3G and 3H).

### miR-155 post-transcriptionally regulated SOCS1 and activated JAK/STAT signaling pathway in human hepatoma cells

**Figure 4:**
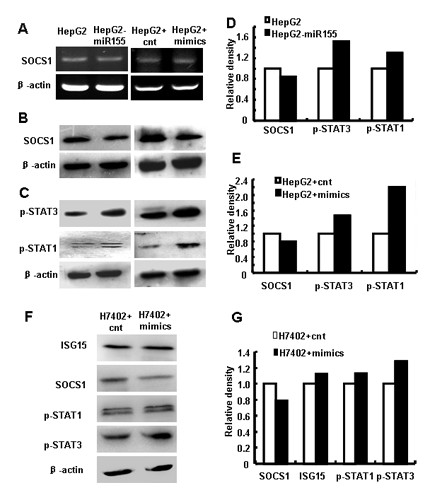
**miR-155 post-transcriptionally inhibited SOCS1 and activated JAK/STAT signaling pathway in human hepatoma cells**. (A) Analysis of SOCS1 mRNA levels by RT-PCR. Total RNA was extracted from HepG2 and HepG2-miR155 cells, and HepG2 cells transfected with 50 nM miR-155 mimics or control RNA. (B) SOCS1, (C) p-STAT1 and p-STAT3 protein levels in cells above were tested by western blot analysis. (D, E) Relative density analysis of the IFN-induced gene protein levels. (F) Western blot analysis of ISG15, SOCS1, p-STAT1 and p-STAT3 protein levels in H7402 cells were performed 48h after transfection. (G) Relative density analysis of the IFN-induced gene protein levels in H7402. One representative of three independent experiments was shown.

Earlier studies indicated that miR-155 and SOCS1 interacted in regulatory T cells, macrophages and human breast cancer cells [18-20]. SOCS1 has been identified to negatively regulate various immune responses and signaling pathways, including the IFN signaling [21,22]. Then we examined whether the translation level of SOCS1 was regulated by miR-155 in human hepatoma cells. Western blot assays indicated that the SOCS1 protein level was reduced significantly in HepG2 cells over-expressing miR-155 compared with the relative control (Figure 4B, D), while the mRNA level of SOCS1 was not obviously changed (Figure 4A).

SOCS1 has been known to inhibit JAK phosphorylation and subsequent binding, phosphorylation and activation of STATs. In the absence of SOCS1, type I IFN-induced STAT1 activation is prolonged, and the antiviral and pro-inflammatory effect of these elements are amplified [21,22]. As SOCS1 is suppressed by miR-155, we speculated that miR-155 over-expression in human hepatoma cells may play a role in JAK/STAT signaling. As expected, p-STAT1 and p-STAT3 were constitutively activated in HepG2 cells over-expressing miR-155 (Figure 4C, E). Similar results were observed in H7402 cells (Figure 4F, G). These findings revealed that miR-155 post-transcriptionally regulated SOCS1 expression and activated JAK/STAT signaling in human hepatoma cells.

### Augmented type I IFN signaling by miR-155 over-expression in human hepatoma cells

**Figure 5:**
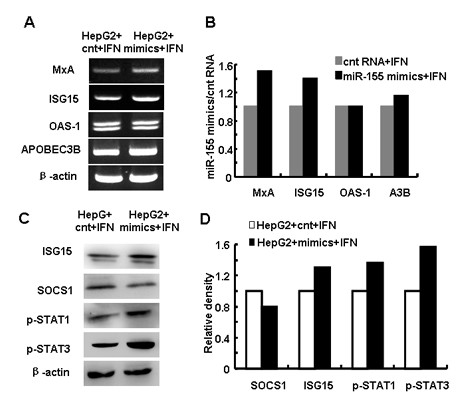
**Augmented type I IFN signaling by miR-155 over-expression in human hepatoma cells**. (A) HepG2 cells transfected with 50 nM miR-155 mimics/control RNA were stimulated by 500 U/mL IFN-α2α for 12 h. RT-PCR analysis was performed to evaluate IFN-inducible genes. (B) Relative density analysis of the IFN-inducible genes. (C) SOCS1, p-STAT1 and p-STAT3 protein levels in cells above were tested by western blot analysis. (D) Relative density analysis of the signaling transduction protein levels in HepG2 cells. One representative of three independent experiments was shown.

We examined type I IFN production in HepG2 cells and found no significant difference in the mRNA levels of either IFN-α or IFN-β when miR-155 was over-expressed (data not shown). We next analyzed the expression of IFN inducible antiviral genes in response to the treatment of IFN-α2α. As shown in Figure 5A and 5B, antiviral genes expressed in HepG2 cells treated with miR-155 mimics were higher than that in control cells. Moreover, the expression of SOCS1 was decreased, while the expression of p-STAT1 and p-STAT3 were increased in HepG2 cells transfected with miR-155 mimics after IFN-α2α treatment (Figure 5C and 5D). These data suggested that over-expression of miR-155 could promote type I IFN signaling and increase IFN-inducible antiviral gene expression in human hepatoma cells.

### Over-expression of miR-155 inhibited HBV in human hepatoma cells

**Figure 6:**
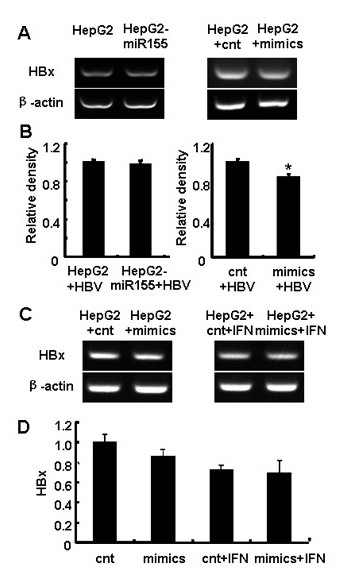
**Ectopic expression of miR-155 inhibited HBV X gene expression in human hepatoma cells**. (A) 0.5 μg PAAV/HBV1.2 plasmid was transfected into HepG2 and HepG2-miR155 cells, or 0.5 μg PAAV/HBV1.2 plasmid and 50 nM miR-155 mimics/control RNA were cotransfected into HepG2 cells. 48 h later, RT-PCR analysis of HBV X gene (HBx) expression was performed. (B) Relative density analysis of HBx expression. (C) 0.5 μg PAAV/HBV1.2 plasmid and 50 nM miR-155 mimics/control RNA were cotransfected into HepG2 cells. 24 h after transfection, cells were treated with IFN-α2α at the concentration of 500 U/mL for another 24 h, and then RNA was extracted and RT-PCR analysis of HBx expression was performed. (D) Relative density analysis of HBx expression for (C). One representative of three independent experiments was shown. (*p < 0.05 by comparison with control cells).

Human hepatitis B virus (HBV) induces hepatitis and is closely associated with the incidence of human hepatoma cancer [9]. HBV was shown to be very efficient at inhibiting the IFN signaling pathway [23-25]. So, we further assessed whether over-expression of miR-155 could attenuate HBV infection. PAAV/HBV1.2 plasmid and miR-155 mimics, or control RNA were cotransfected into HepG2 cells, then expression levels of HBV X gene (HBx) were examined by RT-PCR. As shown in Figure 6A and 6B, HBx level was reduced ~ 12% in HepG2 cells transfected with miR-155 mimics compared with HepG2 cells transfected with control RNA (*P < 0.05*) (Figure 6B right), but there was no significant difference between HepG2 and HepG2-miR155 cells (Figure 6B left). One explanation for this result is that the level of miR-155 in HepG2 cells transfected with miR-155 mimics was much higher than that in HepG2-miR155 cells (Figure 1A and 1B), so we speculated that the antiviral effect of miR-155 depended on its level.

Since miR-155 affect type I IFN signaling pathway, we further addressed whether miR-155 could change the anti-HBV efficiency of exogenous IFN, but as shown in Figure 6C and 6D, the levels of HBV X gene in HepG2 cells treated with miR-155 mimics or control RNA showed no significant difference in the presence of IFN-α2α. Type I IFN can inhibit HBV replication intensively in some HBV-bore cell models based on HBV genome transfection, but miRNA always shows more delicate regulation on virus life cycle than that of siRNA which directly degrade virus RNA. So, IFN-α2α may exert much stronger controlling effects on HBV in hepatocytes than that of miR-155 itself. Taken together, these results indicate that miR-155 exhibited mild anti-HBV effect in human hepatoma cells.

## Discussion

To date, only few publications reveal the roles of miR-155 in viral infection. Viruses can utilize host miRNAs as survival mechanisms, and even encode viral mimics of host miRNAs. One such miR-155 mimic has been found in Kaposi's-sarcoma-associated herpesvirus (KSHV) [7,26,27]. The infection of primary human B lymphocytes with the Epstein-Barr virus (EBV) leads to a sustained elevation of miR-155 expression and contributes to EBV immortalization [28]. These studies suggest that miR-155, encoded by viruses or upregulated by viral infection, contributes to viral-mediated infection via modulating different transcription factors and NF-κB components [14,29-31]. However, a recent research reveals that miR-155 expression is also upregulated by VSV infection in murine macrophages, but the induced miR-155 feedback attenuates the viral replication through enhancing type I IFN signaling by targeting SOCS1 [32]. The biological function of miR-155 in hepatoma cells is still unclear.

HBV is a hepatotropic, enveloped DNA virus, causes acute and always chronic HBV infection and leads to hepatoma fibrosis, cirrhosis and eventually hepatocellular carcinoma [9]. Recent studies have revealed the role of several miRNAs in intricate network of hepatocyte-HBV interactions. Early in 2007, Ai-Guang Guo group firstly predicted that HBV may encode one miRNA [33], but miRBase didn't accept it as a formal member until now. Many host miRNAs were forecasted to bind to both HBV genome and host gene by different laboratories [34-36]. By the end of the year 2010, miR-199a-3p and miR-210 were found to suppress HBsAg expression by directly targeting the HBV S protein coding region and pre-S1 region, respectively [37]; miR125a-5p was then shown to interfere with the viral translation, down-regulating the expression of the surface antigen [38], while miR-1 increases HBV transcription by upregulating farnesoid X receptor α (FXRA), a nuclear receptors binding to the HBV core promoter and regulating HBV transcription and replication. Here, we showed that miR-155 also exhibited mild anti-HBV effect in human hepatoma cells through promoting JAK/STAT signaling pathway and enhancing innate antiviral immunity. Our work reveals a new intricate physiological interplay between miRNAs and HBV replication.

Many evidence shows that miR-155 is over-expressed in a number of neoplastic diseases and plays a significant role in the process of carcinogenesis, acting as an oncomir [39-42]. In this paper, we found that miR-155 expression was very low in HepG2 cells. Though ectopic expression of miR-155 could promote HepG2 cell proliferation, the extent was much weaker than that in other cell lines, such as breast cancer cells [20], which is favorable for miR-155 to be used in antiviral therapeutics without significantly affecting host cell proliferation. These findings indicated that miR-155 play different roles in diverse cells.

In this study, we notice that not all the IFN-induced genes are equally induced by miR-155 over-expression. Since they are all regulated by JAK/STAT signaling and the downstream of type I IFN signaling pathway [43], there must be some other mechanisms to regulate their expression. Though expressions of MxA and ISG15 were elevated by miR-155, their levels were only increase by less than 50% (Figure 3B and 3D), and HBx was only reduced by ~10% (Figure 6B), which is different from a recent report, where VSV replication was largely suppressed by miR-155 in macrophages [32]. This may due to the difference of host cells and viruses. The potency of one miRNA in hepatocytes would be rather weaker than that in lymphocytes, and HBV has its own properties.

Overall, although the anti-HBV effect mediated by miR-155 is lower compared with its anti-VSV effect in macrophages, it might benefit to anti-HBV when combine with other antiviral therapeutics. The detail mechanisms will be further investigated in the future.

## Conclusions

miR-155, as a positive regulator of JAK/STAT signaling by targeting SOCS1, increases the expression of IFN-inducible antiviral genes and enhances innate antiviral immunity in human hepatoma cells. Further more, over-expression of miR-155 exhibited mild anti-HBV effect in human hepatoma cells.

## Materials and methods

### Cell line and cell culture

Human hepatocellular carcinoma cell lines HepG2 and H7402 conserved in our laboratory, were cultured in DMEM (GIBCO/BRL, Grand Island, N.Y. USA) containing 10% FBS in a humidified incubator with 5% CO_2 _at 37°C.

### Vector construction

Total genomic DNA was extracted from HepG2 cells using Genomic DNA Extraction Kit purchased from Sangon Biotech (Shanghai, China) according to the manufacturer's instructions. The miR-155 expression cassette containing the human miR-155 hairpin sequence and flanking regions was amplified from the genomic DNA using the pri-miR-155 primers (Table 1). The cassette was then inserted into an expression vector (pcDNA3). The resulting construct was termed pcDNA3-hsa-miR-155. The construct was confirmed by DNA sequencing. PAAV/HBV1.2 consists of a greater than genome-length HBV fragment that encodes all the proteins of HBV including large, middle and small surface proteins, polymerase protein, X protein, and core and pre-core proteins. It can initiate HBV replication after being transfected into hematoma cell lines [44].

**Table 1:** Primers used for RT-PCR reactions

Transcripts	Product size(bp)	Sequences (5'-3')	Tm(°C)	PCR cycle
β--actin	540	GTGGGGCGCCCCAGGCACCA CTCCTTAATGTCACGCACGATTTC	60°C	28 cycles
SOCS1	301	CACGCACTTCCGCACATTCC TCCAGCAGCTCGAAGAGGCA	55°C	35 cycles
MxA	581	ACCAGCTGAGCCTGTCCGAAGCCC CCGGACCATATCCGTCACGGTG	60°C	35 cycles
ISG15	294	GGTGGACAAATGCGACG ATGCTGGTGGAGGCCCTTAG	60°C	28 cycles
OAS-1	356 454	ACACATTTCAACACAGCCCA TGCAGGTCCAGTCCTCTTCT	60°C	35 cycles
APOBEC3B	981	GTTTGTATCCTGGACCCCCT GAGATGGTGGTGAACGGTCT	60°C	30 cycles
HBx	250	CCGTCTGTGCCTTCTCATCTGC CCTCCGACATCCGTATTTAACCA	60°C	28 cycles
pri-miR-155	307	AGGTGGCACAAACCAGGAA GTTGAACATCCCAGTGACCAG	58°C	38 cycles

### RNA oligonucleotide and cell transfection

miR-155 mimic (dsRNA oligonucleotides), used for the over-expression of miR-155 in hepatoma cells, and negative control RNA (dsRNA oligonucleotides), used as the matched control, were purchased from Shanghai GenePharma Co., Ltd (Shanghai, China). Transfection was performed using Lipofectamine 2000 (Invitrogen, Carlsbad, CA, USA) according to the manufacturer's instructions. For transfection of the RNA oligonucleotides, 50 nM miRNA mimics or control RNA were used.

### Establishment of miR-155 stably over-expressed cells

pcDNA3-hsa-miR-155 plasmid (0.4 μg) were transfected into HepG2 cells, and then 200 μg/mL G418 (GIBCO/BRL) was added 48 h after transfection, and the cells stably over-expressing miR-155 were selected for two weeks until clones form.

### Reverse Transcriptase Polymerase Chain Reaction (RT-PCR) assay

RNA was extracted from HepG2 cells (5 × 10^5^) by Trizol Reagent (Invitrogen, Carlsbad, CA, USA) according to the protocol provided by the manufacturer. The concentration and quality of the extracted RNA were determined by measuring light absorbance at 260 nm (A_260_) at a ratio of (A_260_/A_280_). Total RNA was reverse transcribed to cDNA and standard PCR reaction was performed in a total volume of 25 μl as described previously [45]. The PCR products were electrophoresed on 2% agarose gels and the relative light intensities of bands were analyzed by AlphaEaseFC software. The PCR primers and their product lengths are listed in Table 1 and were synthesized by the Beijing Genomics Institute (Beijing, China).

### Quantitative reverse transcription-PCR assay

The miR-155 level was quantified by quantitative reverse transcription-PCR (qRT-PCR) using specific Bulge-Loop™ miRNA qRT-PCR Primer purchased from Guangzhou Ribobio (Guangzhou, China) with U6 small nuclear RNA as an internal normalized reference.

### Cell proliferation assay

All cellular growth assays were performed in 96-well plates (6 × 10^3 ^cells/well). HepG2 cells were transfected with 50 nM miR-155 mimics or control RNA. Four hours after transfection, equal numbers of viable cells were seeded in 96-well plates and incubated for indicated time. Next, 20 μl MTT (10 mg/ml, Sigma, St Louis, MO, USA) solution was added to each well and the plates were incubated for additional 4 h at 37°C. After centrifugation, MTT solution was then removed, and 200 μl dimethyl-sulfoxide (DMSO; Sigma, St Louis, MO, USA) was added to each well to solubilize the formazan crystals. Absorbance was then read at a wavelength of 570 nm on a scanning multiwell spectrophotometer.

### Western blot

Cells were harvested 48 h after seeded or transfection and homogenized in lysis buffer (30 mM Tris, pH 7.5, 150 mM sodium chloride, 1 mM PMSF, 1 mM sodium orthovanadate, 1% Nonidet P-40, and 10% glycerol) at 4°C, vortexed, and centrifuged at 13,000 g at 4°C for 30 min. The supernatants were mixed in Laemmli loading buffer, boiled for 5 min, and then subjected to SDS-PAGE. After electrophoresis, proteins were transferred onto nitrocellulose membranes and blotted against primary Abs for 12 h. Membranes were washed with 0.1% (v/v) Tween 20 in TBS and incubated with a 1:2000 dilution of HRP conjugated secondary Abs for 1 h. Protein bands were visualized by Immobilon Western Chemiluminescent HRP Sbustrate (Millipore Corporation, Billerica, U.S.A). Antibodies for SOCS1, ISG15 and p-STAT1 were from Cell Signaling Technology (New England BioLabs Inc.); antibody for phospho-STAT3 was from Bioworld Technology, Inc. (Minneapolis, Minnesota, USA); and antibody for β-actin was from Santa Cruz Biotechnology (Santa Cruz, CA, USA).

### Statistical analyzes

All data are expressed as mean±SD and accompanied by at least three distinct experiments. Statistical analysis was performed using SPSS software (version 10.0; SPSS Inc., USA) and *p < 0.05 *were considered statistically significant.

## Competing interests

The authors declare that they have no competing interests.

## Authors' contributions

CS carried out most of the experiments and wrote the manuscript. ZH participated in project design and edit the manuscript. CZ and ZT provided useful advices for the project. JZ is the project leader and was involved in project design, data analysis and finalization of the manuscript. All authors read and approved the final manuscript.
